# Fluoroquinolone Resistance in Drug-Resistant Tuberculosis, Kharkiv, Ukraine, 2019–2023

**DOI:** 10.3201/eid3103.241675

**Published:** 2025-03

**Authors:** Olha Konstantynovska, Tetiana Synenko, Alla Honcharenko, Olha Volobuieva, Tetiana Liadova, Maja Reimann, Christoph Lange, Dumitru Chesov

**Affiliations:** Imperial College London, London, UK (O. Konstantynovska); Kharkiv Regional Phthisiopulmonological Center, Kharkiv, Ukraine (O. Konstantynovska, T. Synenko, A. Honcharenko, O. Volobuieva); V.N. Karazin Kharkiv National University, Kharkiv (O. Konstantynovska, O. Volobuieva, T. Liadova); Leibniz Lung Center, Borstel, Germany (M. Reimann, C. Lange, D. Chesov); German Center for Infection Research (DZIF), Hamburg-Lübeck-Borstel-Riems, Germany (M. Reimann, C. Lange, D. Chesov); University of Lübeck, Lübeck, Germany (C. Lange); Baylor College of Medicine and Texas Children's Hospital, Global Tuberculosis Program, Houston, Texas, USA (C. Lange); Nicolae Testemitanu State University of Medicine and Pharmacy, Chisinau, Moldova (D. Chesov)

**Keywords:** tuberculosis and other mycobacteria, bacteria, Ukraine, fluoroquinolones, respiratory infections, Mycobacterium tuberculosis, antimicrobial resistance, drug resistance, Ukraine

## Abstract

Rifampin-resistant *Mycobacterium tuberculosis* was identified by the World Health Organization as a pathogen of public health critical importance. During 2014–2023, an increase in fluoroquinolone resistance in rifampin-resistant *M. tuberculosis* from Kharkiv, Ukraine, was observed. Efforts to mitigate factors contributing to resistance should be prioritized to prevent further escalation of that threat.

In 2024, the World Health Organization (WHO) officially recognized rifampin-resistant *Mycobacterium tuberculosis* as 1 of 4 antibiotic drug–resistant pathogens of critical global priority (*1*). According to WHO’s 2024 Global TB report, 400,000 persons worldwide develop tuberculosis (TB) caused by a multidrug-resistant/rifampin-resistant (MDR/RR) *M. tuberculosis* (*2*). Of note, the estimated proportion of MDR/RR TB among all new TB cases is 3.2%, whereas among previously treated cases, that figure was 16% (*2*). Regional disparities in the global burden of MDR/RR TB are profound. The greatest incidence is observed in Eastern Europe and Central Asia, where up to 20% of new TB cases and >50% of previously treated cases exhibit MDR/RR TB. Those regions face considerable challenges in implementing effective TB control measures (*2*).

WHO’s 2024 recommendations for drug-resistant TB management included several short treatment regimens, which have shown high efficacy in curing MDR/RR TB. Treatment durations are 6–9 months, which are substantially shorter than the standard 18–20-month regimen (*3*). Eligibility for shorter regimens requires confirmation of drug susceptibility within the regimen. In cases where resistance to agents used in 6- and 9-month regimens is confirmed or suspected, patients must undergo the longer 18-month course. However, emerging resistance to key second-line TB treatment agents in multiple regions threatens the real-world effectiveness of those treatment approaches (*4*).

The objective of this study was to assess resistance rates to second-line TB treatment drugs in the Kharkiv region of Ukraine (2.6 million inhabitants in 2023), which is a country with a high burden of drug-resistant TB, and to compare those findings with our previously reported data from the same region (*5*). We analyzed data from phenotypic drug susceptibility testing, as recorded in the Electronic Register of the TB-Control Program in Kharkiv, for the specified period. Results indicated that 23.1%–31.2% of patients in Kharkiv affected by MDR/RR TB during 2019–2023 had additional resistance to levofloxacin at ≈3-fold greater level than the 10% observed in 2014. Similarly, resistance to moxifloxacin ranged from 10.6% to 20.9% and was the highest rate recorded in the past 2 years, suggesting a notable upward trend in fluoroquinolone resistance (Table). Conversely, resistance to other group A agents (bedaquiline and linezolid) and group B agents (clofazimine and cycloserine) remained low at <1%. We observed high levels of resistance for pyrazinamide (a drug belonging to group C on the WHO drug list), which is a component medicine in the 2024 WHO-recommended 9-month MDR TB regimens. Resistance to pyrazinamide ranged from 54.3% to 58.7% during 2019–2023, compared with 69.6% in 2014.

**Table:** Resistance to second-line drugs in a study of fluoroquinolone resistance in drug-resistant TB, Kharkiv, Ukraine, 2019–2023*

Category	2014	2019	2020	2021	2022	2023	Kendall τ
No. patients with MDR/RR TB	169	333	231	243	155	262	0.07
New TB patients with MDR/RR TB, no. (%)	104 (61.5)	256 (76.9)	178 (77.1)	173 (71.2)	125 (80.6)	187 (71.4)	0.07†
Group A drugs, % resistant							
Moxifloxacin	14.9	NT	15.0	10.6	20.8	20.9	0.6
Levofloxacin	10.0	23.1	27.2	26.9	31.2	28.2	0.73
Bedaquiline	NT	NT	0	0	0.7	0.4	0.55
Linezolid	2.9	0.3	0	0.5	2.7	0.4	−0.07
Group B drugs, % resistant							
Clofazimine	NT	0	0	0	0	0.4	0.63
Cycloserine	5.8	0	NT	NT	NT	NT	NA
Group C drugs, % resistant							
Ethambutol	66.3	75.4	60.1	37.9	49.4	54.4	−0.47
Delamanid	NT	0	1.8	0.9	0.7	1.7	0.2
Pyrazinamide	69.6	54.9	54.9	54.3	55.3	58.6	0
Imipenem/meropenem	NT	NT	NT	NT	NT	NT	NA
Amikacin	23.4	12.4	13.2	18.5	20.4	18.0	0.07
Streptomycin	95.9	75.3	78.4	NT	NT	NT	−0.33
Ethionamide	33.6	32.7	26.1	24.4	27.3	NT	−0.6
Para-aminosalicylic acid	3.1	NT	NT	NT	NT	NT	NA

*Results referred to phenotypic drug susceptibility testing, performed by using the BACTEC MGIT960 culture system (Becton Dickinson, https://www.bd.com), applying World Health Organization–recommended critical concentrations. Drug groups are from the World Health Organization (*3*). MDR/RR, multidrug resistant/rifampin resistant; NA, not applicable; NT, not tested; TB, tuberculosis. †The Kendal τ coeficient for the trend for the percentage of new TB patients over the study period 2014 and 2019–2023 was 0.33.

The substantial increase in fluoroquinolone resistance observed in this study is particularly alarming. Fluoroquinolones play a critical role in MDR/RR TB treatment. Resistance to those agents is a key criterion for defining pre–extensively drug-resistant TB. That resistance is also a noteworthy factor linked to poorer outcomes in patients with MDR/RR TB (*6*). Consequently, because up to 30% of patients in Kharkiv with MDR/RR TB are infected with *M. tuberculosis* strains exhibiting fluoroquinolone resistance, effective TB control faces considerable challenges at times of military oppression. A contributing factor to the rise in fluoroquinolone resistance is likely the insufficient availability or improper use of second-line TB medications (*7*). Our 2014 data indicated deficiencies in MDR/RR TB management at the Kharkiv TB Dispensary, suggesting that the high rates of fluoroquinolone resistance observed during 2019–2023 could be a consequence of suboptimal possibilities for the management of patients with drug-resistant TB in previous years.

In addition to their role in MDR/RR TB treatment, fluoroquinolones are frequently prescribed empirically for common bacterial infections, such as pneumonia and sinusitis. In cases where fluoroquinolones are used as monotherapy for patients with undiagnosed TB, that practice may contribute to bacillary resistance across that drug class (*8*). The resulting symptom relief can delay TB diagnosis, thereby increasing community transmission risk (*9*). In Ukraine, the unregulated use of antibiotic drugs is common practice (*10*), adding to the increase in *M. tuberculosis* resistance to fluoroquinolones. The rapid development of resistance observed here should serve as a cautionary example as new MDR/RR TB drugs, such as bedaquiline, are introduced into clinical settings. Rapid resistance development poses a potential threat to the efficacy of newly implemented TB treatment drugs such as bedaquiline, even if data from this study do not yet reflect such resistance. 

In conclusion, given the ongoing military conflict in the region, heightened vigilance regarding the potential for worsening drug resistance among patients with MDR TB in Kharkiv is essential. Additional efforts to mitigate factors that may contribute to rising resistance should be prioritized to prevent further escalation of the public health threat.
